# Targeted mutagenesis in tetraploid switchgrass (*Panicum virgatum* L.) using CRISPR/Cas9

**DOI:** 10.1111/pbi.12778

**Published:** 2017-08-01

**Authors:** Yang Liu, Paul Merrick, Zhengzhi Zhang, Chonghui Ji, Bing Yang, Shui‐zhang Fei

**Affiliations:** ^1^ Interdepartmental Graduate Major in Plant Biology Iowa State University Ames IA USA; ^2^ Department of Horticulture Iowa State University Ames IA USA; ^3^ Interdepartmental Graduate Major in Genetics and Genomics Iowa State University Ames IA USA; ^4^ Department of Genetics, Development and Cell Biology Iowa State University Ames IA USA

**Keywords:** CRISPR/Cas9, gene editing, switchgrass, tillering, transient assay

## Abstract

The CRISPR/Cas9 system has become a powerful tool for targeted mutagenesis. Switchgrass (*Panicum virgatum* L.) is a high yielding perennial grass species that has been designated as a model biomass crop by the U.S. Department of Energy. The self‐infertility and high ploidy level make it difficult to study gene function or improve germplasm. To overcome these constraints, we explored the feasibility of using CRISPR/Cas9 for targeted mutagenesis in a tetraploid cultivar ‘Alamo’ switchgrass. We first developed a transient assay by which a non‐functional green‐fluorescent protein gene containing a 1‐bp frameshift insertion in its 5′ coding region was successfully mutated by a Cas9/sgRNA complex resulting in its restored function. *Agrobacterium*‐mediated stable transformation of embryogenic calli derived from mature caryopses averaged a 3.0% transformation efficiency targeting the genes of *teosinte branched 1(tb1)a* and *b* and *phosphoglycerate mutase* (*PGM*). With a single construct containing two sgRNAs targeting different regions of *tb1a* and *tb1b* genes, primary transformants (T0) containing CRISPR/Cas9‐induced mutations were obtained at frequencies of 95.5% (*tb1a*) and 11% (*tb1b*), respectively, with T0 mutants exhibiting increased tiller production. Meanwhile, a mutation frequency of 13.7% was obtained for the *PGM* gene with a CRISPR/Cas9 construct containing a single sgRNA. Among the *PGM* T0 mutants, six are heterozygous and one is homozygous for a 1‐bp deletion in the target region with no apparent phenotypical alterations. We show that CRISPR/Cas9 system can generate targeted mutagenesis effectively and obtain targeted homozygous mutants in T0 generation in switchgrass, circumventing the need of inbreeding.

## Introduction

Mutants are critical for determining gene function and elucidating metabolic pathways and can be a valuable resource for crop improvement as well. Since 1980s, research on methods for generating gene knockout mutants had gained much attention (Jiang *et al*., 2013). In recent years, targeted gene editing technologies such as zinc finger nucleases (ZFNs) and TAL effector nucleases (TALENs) have been widely used in animal and plant systems (Beerli and Barbas, 2002; Char *et al*., 2015; Li *et al*., 2012; Nicolia *et al*., 2015; Townsend *et al*., 2009; Urnov *et al*., 2005, 2010; Wang *et al*., 2014). Since 2013, the clustered regularly interspaced short palindromic repeats (CRISPR) and CRISPR‐associated protein (*cas*) system have become a powerful tool for targeted genome editing, requiring only a single protein and a programmable guide RNA (Jinek *et al*., 2012). CRISPR are tandem arranged prokaryotic direct repeat DNA sequences containing dyad symmetry, with interspersed non‐repeating spacer sequences. Together with the *cas* genes, CRISPR/Cas functions in prokaryotic acquired immunity against foreign bacteriophage and plasmid DNA invasion through RNA‐guided endonuclease digestion (Jinek *et al*., 2012).

In the most widely used type II CRISPR/Cas9 system derived from *Streptococcus pyogenes*, a trans‐encoded crRNA (tracrRNA) was discovered, which mediates pre‐crRNA maturation (Deltcheva *et al*., 2011), and was found to be necessary for crRNA's association with the Cas9 endonuclease and, therefore, foreign DNA interference (Jinek *et al*., 2012). Cas9 had also been shown to be the only Cas protein necessary for protospacer cleavage in the type II system (Barrangou *et al*., 2007; Garneau *et al*., 2010; Jinek *et al*., 2012; Sapranauskas *et al*., 2011). The type II CRISPR/Cas9 system makes double‐strand breaks (DSBs) just upstream of a protospacer adjacent motif (PAM) of three nucleotides long (NGG). The DSBs can be repaired by either the error‐prone non‐homologous end‐joining (NHEJ) repair pathway, which generates mutants, or homologous recombination (HR) repair pathway, which, depending on the type of editing template provided, may also generate mutants (Joung and Sander, 2012; Svitashev *et al*., 2015). Because CRISPR/Cas9 works in *trans*, a mutation can be created at a locus distant from the CRISPR/Cas9 transgene insertion site. Through traditional breeding, the transgene can be eliminated without affecting the mutation (Xu *et al*., 2015a, b). Such mutants are very different from traditional transgenic plants and may require less or no regulatory oversight. Recently, DNA‐ and selectable marker‐free mutant plants have been created by delivery of preassembled Cas9‐sgRNA ribonucleoproteins into maize (Svitashev *et al*., 2015) and wheat (Liang *et al*., 2017) using particle bombardment.

CRISPR/Cas9‐based genome editing technology is highly versatile. Because there are usually abundant PAM (NGG) sequences in most genes, numerous single‐guide RNAs (sgRNAs) can be readily designed and synthesized for targeting any genes. Since the creation of the first programmable CRISPR molecular tool (Jinek *et al*., 2012), various CRISPR/Cas9‐based tools have been developed for genome editing in mammals and plants (Hwang *et al*., 2013; Jiang *et al*., 2013; Ma *et al*., 2015; Xie *et al*., 2015). With multiplexed Cas9‐sgRNAs, each sgRNA targeting a different gene, multiple genes can be edited with a single facile CRISPR/Cas9 construct. It is also feasible to remove gene clusters with large chromosomal deletions induced by Cas9/sgRNAs (Xie *et al*., 2015; Zhou *et al*., 2014). Inheritance of mutations created by CRISPR/Cas9 has been demonstrated in Arabidopsis, rice and tomato (Feng *et al*., 2014; Ito *et al*., 2015; Ma *et al*., 2015). By providing a repair template, point mutations can be introduced into endogenous genes (Fauser *et al*., 2014; Li *et al*., 2015; Mao *et al*., 2013; Sun *et al*., 2016; Svitashev *et al*., 2015), and when a template DNA containing the desirable mutations is provided, such mutations can be introduced into a specific locus of the plant's genome through homologous recombination, creating a genuine gene replacement mutant (Li *et al*., 2015; Schiml *et al*., 2014; Svitashev *et al*., 2015).

Switchgrass is high yielding because of its highly efficient C4 photosynthesis system. It also grows well on marginal land, which, along with its perenniality, makes it ideal for producing lignocellulose‐based biofuel (Nageswara‐Rao *et al*., 2013). It was named a model bioenergy crop by the U.S. Department of Energy in 1991 (Wright and Turhollow, 2010). Extensive research on genetic diversity, genome structure, genetic mapping and gene function on switchgrass has been conducted during the past decades (Lu *et al*., 2013; Okada *et al*., 2010; Rinerson *et al*., 2015; Xu *et al*., 2015a,b). However, because switchgrass is highly self‐incompatible (Martínez‐Reyna and Vogel, 2002) and is predominantly tetraploid or octoploid (Hopkins *et al*., 1996; Hultquist *et al*., 1996, 1997), it is difficult to conduct forward or reverse genetic analyses in switchgrass and inbred lines are difficult to develop. Therefore, the development of an effective molecular tool to assist in the development of improved switchgrass cultivars is urgently needed. It has been shown that CRISPR/Cas9 can generate targeted mutation in polyploid plants such as wheat and potato (Shan *et al*., 2013; Wang *et al*., 2014, 2015). Yet, no report on the use of CRISPR/Cas9 in switchgrass has been published. In the present study, we established a transient assay protocol in switchgrass using mesophyll protoplasts and a non‐functional GFP to validate CRISPR/Cas9 activity. Furthermore, we showed that CRISPR/Cas9 is effective to simultaneously create targeted mutations in both *teosinte branched 1(tb1)a* (Pavir.Ia00838) and *b* (Pavir.Ib04362), and in the *Phosphoglycerate mutase* (*PGM*, Pavir.Da00700) gene for which a homozygous mutant was obtained in the primary transformant (T0), bypassing the need of further crossing or inbreeding.

## Results

### Cas9/sgRNA is capable of producing precise mutations in switchgrass protoplasts

**Figure 1:**
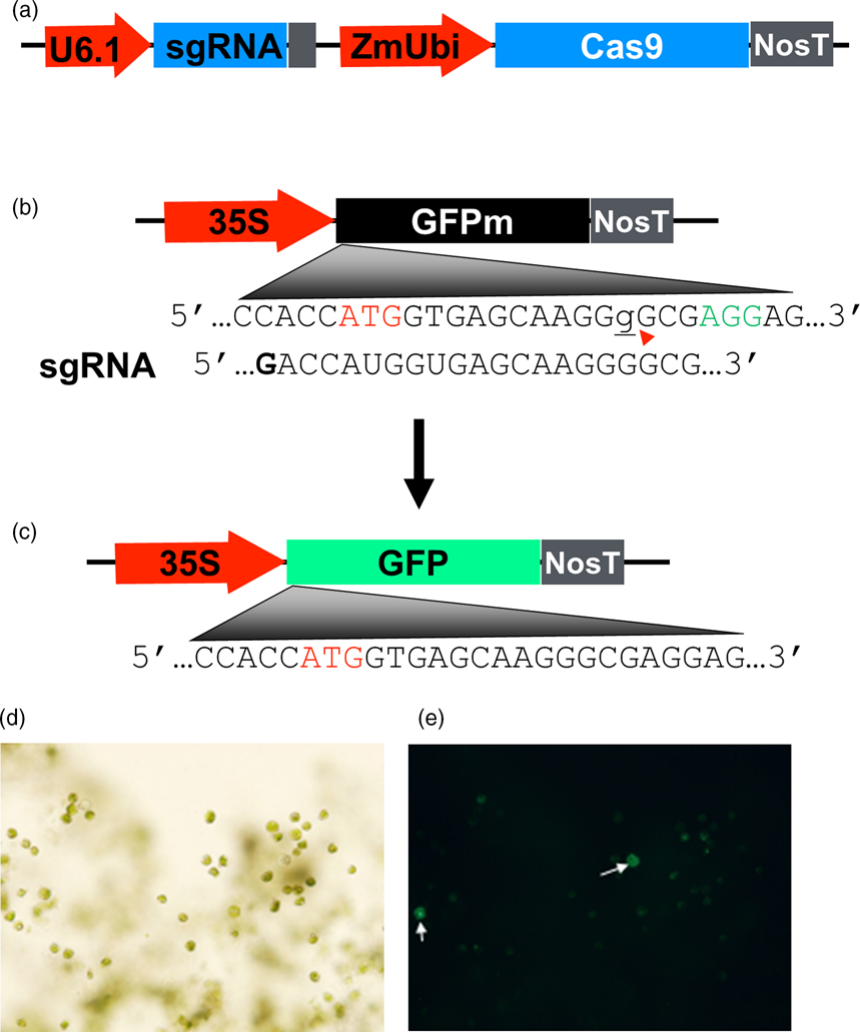
Switchgrass protoplast system for assessing CRISPR/Cas9 activity with the *GFP* reporter gene. (a) A schematic of the CRISPR/Cas9 construct expressing a single‐guide RNA (targeting the mutated *GFP* gene in this case) under a rice U6 promoter and a rice codon‐optimized *Cas9* under the maize ubiquitin gene promoter. (b) A construct contains the 35S promoter, a non‐functional *GFP* gene (*GFPm*) and a NOS terminator. *GFPm* contains an insertion mutation with a guanine (the lower case, underlined letter g) that is located downstream of the translation start site (ATG in red) and three nucleotides upstream of the PAM sequence (AGG in green) within the target site for sgRNA. The red arrow head indicates the presumptive sgRNA/Cas9 cleavage site. (c) Schematics of the restored, functional *GFP* in which the inserted guanine is deleted. (d) and (e) Protoplasts transfected with *GFPm* and sgRNA/Cas9 result in some protoplasts (indicated by arrows) emitting green fluorescence. Paired images are of the same protoplasts, taken using a Nikon Eclipse E200 microscope with 20× objective, bright field (d) and fluorescence (e).

To investigate whether the highly efficient CRISPR/Cas9 established in rice (Zhou *et al*., 2014) is also capable of inducing site‐specific mutations in switchgrass, we first established an efficient protocol for protoplast isolation and transfection using switchgrass mesophyll cells; using the protoplast system, we tested the activity of the Cas9 and sgRNA on the GFP reporter gene when co‐expressed in the same protoplasts. The CRISPR/Cas9 system consisting of the rice codon‐optimized Cas9 gene under the maize ubiquitin gene promoter, and a rice U6 promoter to express the sgRNAs was previously described (Figure 1a, Zhou *et al*., 2014). The GFP reporter construct was made with a non‐functional GFP gene (GFPm) that contains a 1 nucleotide (G) insertion downstream of translation start site under the CaMV 35S promoter (Figure 1b). The frameshift mutation abolishes the ability of the gene to produce a fluorescent signal. When a sgRNA targeting the GFP mutation site, Cas9 and GFPm are in a single switchgrass protoplast, DSB at the mutation site in GFPm occurs and DNA repair through NHEJ leads to new mutations (insertions/deletions), some of which correct the reading frame of the gene, restoring the GFP gene function and emitting green fluorescence (Figure 1c).

The plasmid DNA of p35S:GFPm, pU6:gRNA_GFP and pZmUbi:Cas9 was cotransferred into switchgrass protoplasts. Fluorescence signal was detected about 60 h after the cotransformation and about 3.3% of the protoplasts displayed strong green fluorescence signals, similar to the level observed in protoplasts that were transformed with the functional GFP gene under a rice ubiquitin gene promoter as a control (Figure 1d,e). In the control, about 25% of the viable protoplasts showed strong green fluorescence signals 24 h after transformation (data not shown). The time lag in detecting the green fluorescence may reflect the time required for the CRISPR/Cas9 to induce mutations and produce GFP. As another control, protoplasts transformed only with the non‐functional GFP gene all failed to produce fluorescent signals (data not shown). Together, these experiments demonstrated that CRISPR/Cas9 system is able to induce targeted mutations in switchgrass protoplasts.

### CRISPR/Cas9 is capable of inducing mutations in endogenous genes in switchgrass

To test whether the CRISPR/Cas9 system is capable of introducing mutations at target genomic loci, three genes, *teosinte branched 1(tb1)a*,* b* and *phosphoglycerate mutase* (*PGM*), were chosen for targeted mutagenesis. *tb1* is a well‐studied key regulator of branch architecture in maize and its homologs are shown to function similarly in other grasses (Whipple *et al*., 2011). With disruption of *tb1* function, plants typically show an easily observable bushy phenotype. There are two *tb1* genes, *tb1a* and *tb1b* in switchgrass with 90% amino acid identities between them. Our CRISPR/Cas9 constructs are capable of generating either single or double mutants for the *tb1a* and *b* genes so that we can characterize the function for each gene in case there is functional redundancy between them. The *PGM* gene encodes the enzyme of phosphoglycerate mutase (PGM), which catalyses the reversible step of converting 3‐phosphoglycerate to 2‐phosphoglycerate in the glycolysis pathway (Jedrzejas *et al*., 2000). There are two *PGM* genes in Arabidopsis, and double mutants for the *PGM* genes showed severely impaired growth, failure to produce pollen, and defects in the energy‐requiring processes of stomatal movements (Zhao and Assmann, 2011).

These three genes were isolated and confirmed for sequence identities from the switchgrass cultivar ‘Alamo’, a lowland tetraploid based on sequence information available at Phytozome (www.phytozome.net, verified on April 21, 2017). To observe the phenotype of mutants, it is often necessary to create mutants homozygous for the mutated gene. Switchgrass is a polyploid and is naturally cross‐pollinated; therefore, individuals may be highly heterozygous with possible multiple alleles for each gene, which could present a problem for CRISPR/Cas9 to create mutants with all alleles knocked out. This is because a single sgRNA may not be able to recognize the different alleles of a gene due to sequence mismatch. To address this issue, we chose target sequences from regions of a gene that do not vary, that is without single nucleotide polymorphism (SNP) among alleles based on existing sequence database and confirmed sequences.

**Figure 2:**
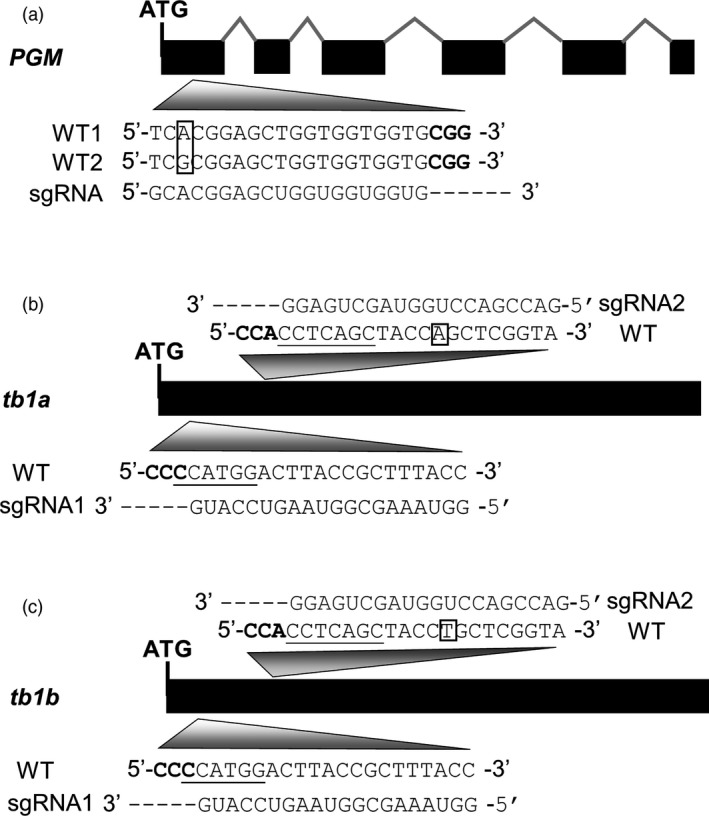
Schematics of gene structures with exons (solid, black bars), introns (‘^’ lines) and sequences of target sites and guide RNAs. (a) *PGM* gene with two different alleles; boxed letters indicate the allelic SNP; PAM sequence is in bold. (b) and (c) Gene structures of *tb1a* and *tb1b* and corresponding guide RNAs. Boxed letters indicate the SNP between the two genes; sequences complementary to PAM are in bold. Underlined sequences indicate enzyme recognition sites used for mutant allele enrichment.

A target site in the first exon, 41‐bp downstream of the translation start site, was chosen in the *PGM* gene for design of the guide RNA gene (Figure 2a). Meanwhile, two target sites from two exon regions conserved between *tb1a* and *tb1b* were selected for design of two guide RNA genes for targeted mutagenesis. One target site is located at 26‐bp downstream of the translation start sites in both genes, and the other target site is located 150‐bp downstream of their translation start sites (Figure 2b,c). Frameshift mutations downstream the translation start site will lead to the large truncations of predicted peptides for the genes (e.g. *PGM*,* tb1a* and *tb1b*) and consequently the gene knockouts. To test the efficiency and specificity of the CRISPR/Cas9 system, one sgRNA has a perfect match in *tb1a* and *tb1b*, while another sgRNA has sequence perfectly matching the *tb1a* target region but has a 1‐bp mismatch with the *tb1b* target region. Because the G nucleotide is the preferred transcription initiation site for U6 promoter (Shan *et al*., 2013), the original first nucleotide T of first target sequence for *tb1a* and *b* and *PGM* genes was replaced by G (Figure 2a–c).

pENTR4:gRNA4 derived from pENTR™ was used as the entry vector, which can accommodate two chimeric sgRNAs, each under the control of a different rice U6 promoter (Figure 1a; Figure S1). The destination vector pUbi‐Cas9 derived from a Gateway^®^ cloning system contains a rice codon‐optimized *Cas9*, which is driven by the maize ubiquitin gene promoter and terminated by the nopaline synthase (NOS) gene terminator region (Figure 1a; Figure S2, Zhou *et al*., 2014). This vector contains the hygromycin phosphotransferase (*hpt*) gene which confers resistance to hygromycin and can therefore be used for selecting transgenic plants. Through LR reaction, sgRNA, either single in the case of the *PGM* gene, or two sgRNAs that target the *tb1a* and *tb1b* genes were mobilized into the *Cas9* and *hpt* gene containing binary vector for genetic transformation and targeted mutagenesis.

### Sequence characterization of putative CRISPR/Cas9‐induced mutants

A total of 2428 pieces of calli infected with *Agrobacterium* were subjected to selection with hygromycin and 73 independently transformed calli successfully regenerated functional plants, resulting in an average of 3.0% transformation efficiency (number of independent events / number of starting calli on selection medium).

**Figure 3:**
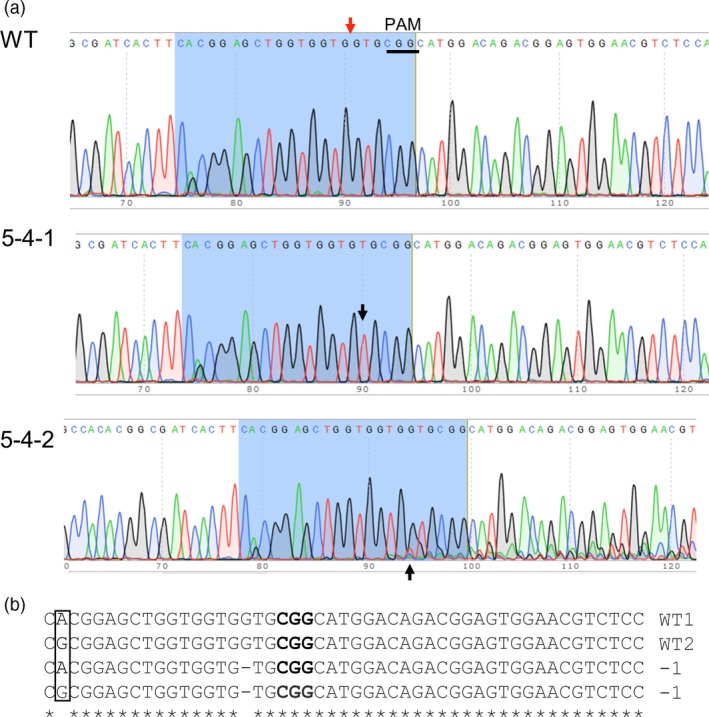
Representative sequencing results of the target regions within the *PGM* gene. (a) Chromatograms of DNA sequences in wild type (WT), homozygous mutant (5‐4‐1) and heterozygous mutant (5‐4‐2). The red arrow indicates the CRISPR cleavage site, and the PAM sequence is underlined in WT; black arrow head in 5‐4‐1 points to the deletion of G relative to the wild‐type *PGM* sequence; black arrow head in 5‐4‐2 points to double peak in presence of G and T, suggesting the deletion of G in some PCR products. (b) Alignment of partial sequences of different alleles of the *PGM* gene spanning the target region obtained by colony sequencing of PCR products of a heterozygous and the homozygous mutant plant, along with the wild‐type control. PAM sequence is in bold. The bases A and G highlighted in the box show the presence of an allelic SNP within the target region wild type.

For the *PGM* gene, the sgRNA‐PGM/Cas9 construct with one sgRNA induced mutation at a frequency of 13.7% (Table 1). Among the seven *PGM* mutant plants, six are heterozygous mutants with at least one mutant allele and a wild‐type allele and one plant is a homozygous mutant with only the mutant allele. Mutants containing heterozygous mutations in the target regions were discovered by the presence of small double peaks in the chromatogram that is absent from the wild type (Figure 3a). Because the double peaks observed in the heterozygous mutant plants were small, yet not seen in sequences of the wild‐type plants, the PCR amplicons used for sequencing were cloned and multiple clones were subjected to sequencing again to confirm the nature of the heterozygous mutations. Sequencing of individual clones revealed that a single‐base pair deletion is present in one of eight randomly selected colonies derived from the heterozygous mutant plant 5‐4‐2; other heterozygous mutant plants were shown to have the same type of mutation (Figure 3b). Given the small size of the double peaks and the results from the cloning and sequencing experiments, it is likely that only a single allele has been mutated in the heterozygous mutants. Similarly, the PCR amplicons used for sequencing from the homozygous mutant plant containing a 1‐bp deletion were also cloned and resequenced. Sequencing of nine individual clones confirmed the presence of the same single‐base pair deletion, indicating all alleles were mutated at the target site (Figure 3b).

**Table 1:** Frequencies of CRISPR/Cas9‐induced mutations for *tb1a*,* tb1b* and *PGM* genes

Gene	Callus line number^a^	Number of sequenced independent transgenic events	Number of plants sequenced	Number of mutated plants	Mutation frequency (%)
*tb1*	7	32	46	44 (*tb1a*)	95.6
				5 (*tb1b*)^b^	11
*PGM*	1	1	3	0	13.7
	3	1	19	5	
	5	8	25	2	
	13	4	4	0	

a Each individual callus line was derived from a single caryopsis.

b *tb1b* mutant plants also carry the *tb1a* mutations.

Because the cultivar Alamo is a tetraploid, mutants may escape detection by PCR if the mutation occurs only in one or two alleles. To increase the power of detecting mutant alleles, genomic DNA samples from putative mutants were digested with a restriction enzyme (a recognition site for the restriction enzyme spans the target sites of *tb1* genes, Figure 2b,c) to enrich mutated alleles followed by PCR amplification with primers flanking the target sites. PCR amplicons from such enriched DNA from a total of 46 plants were sequenced and a mutation rate for *tb1a* target site was 95.6% (44 out of 46 plants), whereas a mutation rate of 11% (five of 46 plants) was obtained for the *tb1b* gene (Table 1). These five plants contain mutations for both the *tb1a* and *tb1b* genes, indicating that a single CRISPR/Cas9 with two sgRNAs can simultaneously mutate two different genes with high homology in switchgrass.

**Figure 4:**
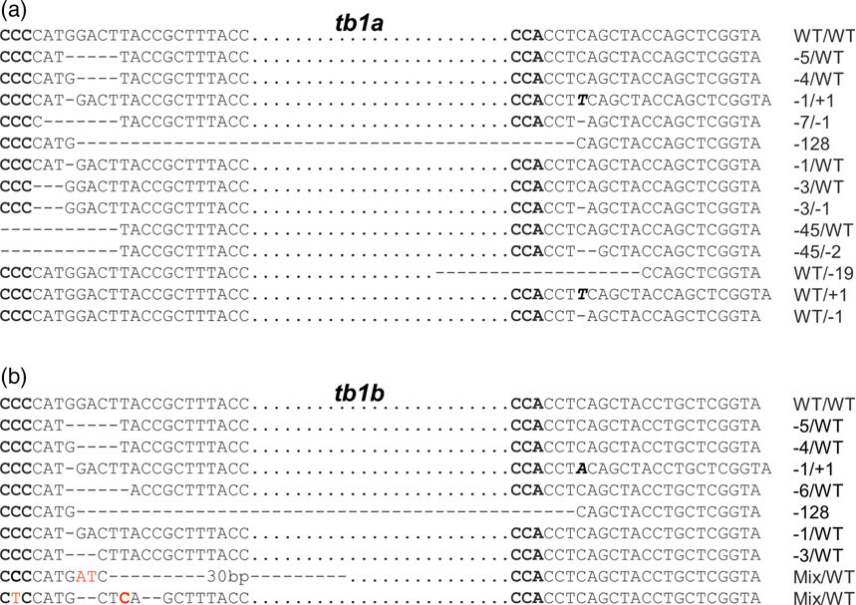
Representative sequences of *tb1* mutations induced by CRISPR/Cas9 with deletions (dashed lines), insertions (italic, bold letters) and substitutions (red letters). (a) and (b) are for mutations of *tb1a* and *tb1b*, respectively. Sequences complementary to PAM sequence are in bold. Sequences between two target sites are indicated by black dots.

**Figure 5:**
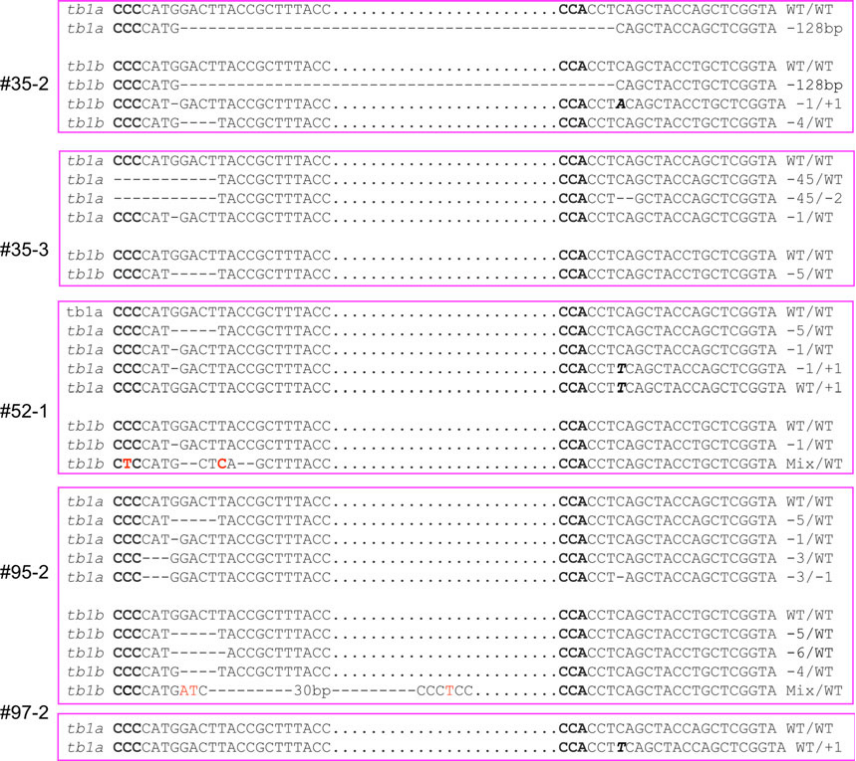
Sequences of alleles from *tb1a* and *tb1b* mutants selected for phenotypic characterization. Mutations induced by CRISPR/Cas9 are deletions (dashed lines), insertions (italic, bold letters) and substitutions (red letter). Sequences complementary to PAM sequence are in bold. Sequences between two target sites are indicated by black dots.

Each of the *tb1* mutants contained at least two types of alleles (Table 2; Figure 5). For the *tb1a* gene, 13 randomly selected mutants from independent transformed events #19 (1 plant), #24 (1 plant), #26 (1 plant), #30 (2 plants), #35 (2 plants), #52 (1 plant), #65 (1 plant), #75 (1 plant), #90 (1 plant), #95 (1 plant) and #97 (1 plant) were genotyped at the two target regions. Of the forty‐six mutated alleles, thirteen types of mutations were found, most of which were deletions ranging from 1 bp to 128 bp (Figure 4a, Table 2). There were eight types of mutated alleles with a mutation at one target site only (A, B, C, D, E, F, G and H, Table 2), while the remaining five types contained mutations at both target sites within the *tb1a* gene (I, J, K, L and M, Table 2). There were four mutant plants containing a large 128‐bp deletion between the two target sites (Table 2).

**Table 2:** Types and frequencies of CRISPR/Cas9 induced mutations for *tb1a*,* tb1b* and *PGM* genes

Target gene	Mutation type (designation)	Plants with the mutation (the first number represents the event number, while the second number represents individual plants derived from the event)	Number of sequenced colonies carrying the mutation	Frequency (No. of colonies with specific mutations/No. of total colonies with mutations) (%)
*tb1a*	First site: 1‐bp deletion (A)	30‐3, 35‐3, 52‐1, 65‐2, 90‐3, 95‐2	8	17.4
	First site: 3‐bp deletion (B)	95‐2	4	9
	First site: 4‐bp deletion (C)	30‐1	1	2
	First site: 5‐bp deletion (D)	30‐1, 30‐3, 52‐1, 65‐2, 95‐2	7	15.2
	First site: 45‐bp deletion (E)	24‐1, 35‐3, 75‐1	5	11
	Second site: 1‐bp deletion (F)	52‐1	1	2
	Second site: 19‐bp deletion (G)	19‐5	2	4
	Second site: 1‐bp insertion (H)	97‐2, 52‐1	5	10.9
	First site: 3‐bp deletion and second site: 1‐bp deletion (I)	95‐2	1	2
	First site: 7‐bp deletion and second site: 1‐bp deletion (J)	90‐3, 26‐1	3	6.5
	First site: 45‐bp deletion and second site: 2‐bp deletion (K)	35‐3	1	2
	First site: 1‐bp deletion and second site: 1‐bp insertion (L)	52‐1	4	9
	128‐bp deletion between two sites (M)	30‐3, 30‐1, 35‐2, 90‐3	4	9
Subtotal			46	100
*tb1b*	First site: 1‐bp deletion (N)	11‐2, 35‐2, 52‐1	5	25
	First site: 3‐bp deletion (O)	35‐2	1	5
	First site: 4‐bp deletion (P)	95‐2, 35‐2	2	10
	First site: 5‐bp deletion (Q)	35‐3, 95‐2	2	10
	First site: 6‐bp deletion (R)	95‐2	3	15
	First site: 30‐bp deletion and substitution (S)	95‐2	1	5
	First site: 1‐bp deletion and second site: 1‐bp insertion (T)	35‐2	1	5
	First site: deletion, insertion and substitution, second site: 1‐bp insertion (U)	52‐1	3	15
	128‐bp deletion between two sites (V)	35‐2	2	10
Subtotal			20	100
*PGM*	1‐bp deletion	3‐1, 3‐2, 3‐6, 3‐7, 3‐9, 5‐4‐1 and 5‐4‐2	20	100

For the *tb1b* gene, sequencing results from the five mutated plants revealed nine types of mutations (Table 2; Figure 4b). There were five types of mutations with deletions ranging from 1 bp to 6 bp at the first target site only (N, O, P and R) with the remaining mutants carrying deletions/insertions at both target sites (S, T and U) (Table 2). Similar to *tb1a*, the same large 128‐bp deletion between the two target sites for *tb1b* was observed in the mutant plant 35‐2. Interestingly, individual plants derived from the same transgenic event carried different mutations. For example, the large 128‐bp deletion was only found in the mutant plant 35‐2, but not in 35‐3, which was also derived from the transgenic event 35 (Figure 5, Table 2).

### Morphological characterization of mutant plants

Four plants (35‐2, 35‐3, 52‐1 and 95‐2) containing mutations in both *tb1a* and *tb1b* and one plant (97‐2) carrying a mutation only in *tb1a* were phenotypically characterized. All of the mutated plants contained mutated alleles with frameshift mutations for the *tb1a* and *tb1b* genes with the only exception of the mutant 97‐2 (Figure 5). Plant 95‐2 also contains the in‐frame 3‐bp and 6‐bp deletions, in addition to the frameshift mutation. Mutants 95‐2 and 52‐1 contained more than four distinct mutant alleles of *tb1a* (Figure 5), suggesting they are likely chimeric plants.

**Figure 6:**
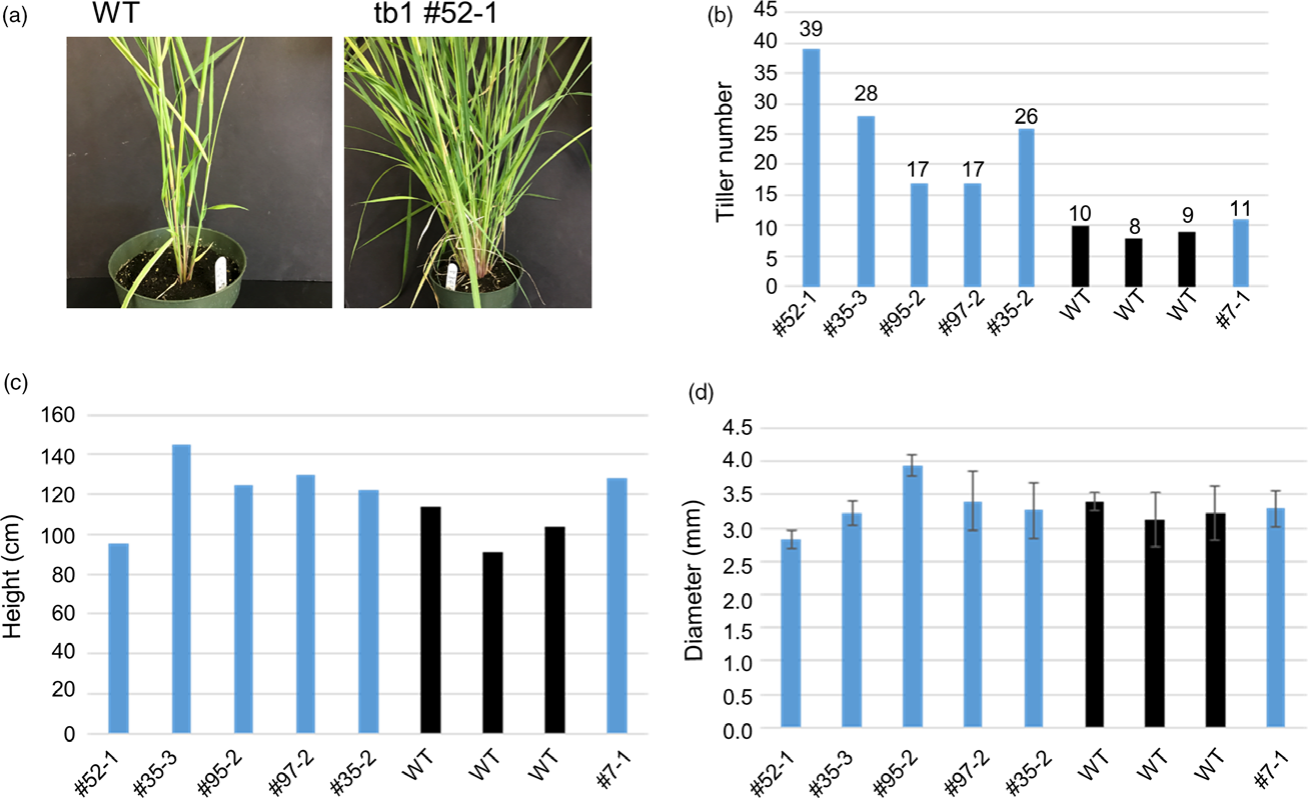
Morphological characterization of wild type (WT‐1, 2 and 3), non‐mutant transgenic plant (#7‐1) and mutants (#52‐1, 35‐3, 95‐2, 97‐2 and 35‐2). (a) Representative plants of wild type (WT) and one mutant line with increased tiller numbers (*tb1* #52‐1) are shown. (b) Tiller numbers for different lines. (c) Average plant height for different lines. (d) Average stem diameter for different lines. Error bars indicate the SD.

The three mutated plants (52‐1, 35‐3 and 35‐2) possessing mutated *tb1a* and *tb1b* alleles all showed a large increase in tiller production compared to the wild‐type plants and a transgenic, non‐mutant plant (Figure 6) at the beginning of the reproductive stage. The mutant 52‐1 produced 39 tillers while mutant plants 35‐2 and 35‐3 produced 26 and 28 tillers, respectively, more than twice of that observed for the wild‐type plants. Mutant 97‐2 with a frameshift mutant allele (1‐bp insertion) in *tb1a* only also showed an increase in tiller numbers compared to the wild‐type plants, but the increase is less than that observed in plants containing both *tb1a* and *tb1b* mutated alleles (Figure 6) with the exception of mutant 95‐2, which, despite having mutations at both *tb1a* and *tb1b* target regions, produced the same tiller number as the mutant 97‐2. It is worth noting that mutant 95‐2 contained in‐frame mutations in addition to frameshift mutation. Based on these results, switchgrass *tb1a* and *tb1b* genes may negatively regulate branching redundantly in a dosage‐dependent manner. These mutant plants have similar height and tiller diameters, suggesting that knockout of *tb1* genes does not impact tiller growth except till number (Figure 6).

## Discussion

Protoplast‐based transient gene expression assay is a valuable approach to assessing the activity of CRISPR/Cas9 constructs in site‐directed mutagenesis *in vitro* before embarking on conducting the more time‐consuming, expensive stable transformation. Similar approach has been successfully used in wheat, rice and potato protoplasts to determine the effectiveness of CRISPR/Cas9 or TALEN constructs (Nicolia *et al*., 2015; Shan *et al*., 2013). In the present study, for the first time we developed a protoplast isolation and transient assay protocol to measure the effectiveness of CRISPR/Cas9 constructs with a reporter system in switchgrass. The readout of the *GFP* reporter system reflects the ability of CRISPR/Cas9 to cause DSBs at the target site and the efficiency of cellular DNA repair through NHEJ to correct the frameshift mutation in the non‐functional *GFP* gene and emit fluorescence signal in single switchgrass protoplasts.

Despite the presence of an apparent mutation, sequencing PCR amplicons from DNA extracted from the pooled protoplasts including those emitting fluorescence signals failed to reveal any CRISPR/Cas9‐induced mutations in the target sites. This is most likely due to the low percentage of protoplasts carrying the anticipated mutated non‐functional *GFP* gene; that is, only 3.3% of protoplasts carries the functionally restored *GFP* gene. To detect low‐frequency mutations in a protoplast population, digestion of the pooled DNA with a restriction enzyme that recognizes a cutting site spanning the Cas9 cleavage site in wild‐type sequences is usually required to enrich the mutated DNA molecules before PCR amplification (Wang *et al*., 2014). Unfortunately, because our expected Cas9 cleavage sites do not contain a restriction enzyme recognition sequence, we were unable to use restriction enzyme to enrich the mutated transferred DNA, which might explain why we failed to detect the expected mutations.

In stably transformed switchgrass, of the three genes (*tb1a*,* tb1b* and *PGM*) tested, mutation efficiencies were 95.6%, 11% and 13.7%, respectively. Because mutation frequency of *tb1b* is much lower than *tb1a*, it is likely that CRISPR/Cas9 system may have sequence preference for targeted mutagenesis. At the *PGM* target site, only one‐base pair deletion was found in all mutants, while at the *tb1a* and *tb1b* target sites, thirteen and nine types of mutations were detected, respectively. Insertion mutations were only found at the second target site in *tb1* genes. These results suggest that the type of target gene may affect the type of mutations and their frequencies. However, because the number of mutated *PGM* plants was small, the correlation between the sequence of the target genes and mutation types needs further investigation.

Despite their very different chromosomal locations, *tb1a* and *tb1b* can probably be viewed as non‐positional homeologs, resulting from the same ancestral gene following chromosome rearrangements during speciation or polyploidization (Glover *et al*., 2016). However, both *tb1a* and *tb1b* in switchgrass appear to have generated their own positional homeologs during polyploidization. Sequencing of these homeoalleles, however, revealed near‐identical sequences. For example, for *tb1a*, double peaks are present in the relevant PCR amplicons only at 600‐bp downstream of transcription starting site, which is known to harbour a SNP (G/C, http://www.phytozome.net), and no sequence variation was detected for *tb1b* despite a G/A SNP that is known to exist at 634‐bp downstream of transcription starting site (http://www.phytozome.net). This high sequence identity between homeoalleles is unusual given that switchgrass is an obligate open‐pollinated species and the lowland switchgrass ecotypes including Alamo are suggested to be an allotetraploid (Serba *et al*., 2013). However, this high level of sequence conservation may highlight the important roles that *tb1a* and *tb1b* play in switchgrass growth and development.

In the current study, among the 23 types of mutated alleles induced by CRISPR/Cas9 from three genes, there were only four insertion mutations and two substitution mutations. This is different from other species such as rice, Arabidopsis and wheat where many insertions were reported (Ma *et al*., 2015; Wang *et al*., 2014). This is likely caused by the unique intrinsic DNA repair mechanisms present in switchgrass. In detailed studies from rice and Arabidopsis, the majority of deletions resulting from CRISPR/Cas9 were less than 10 bp (Zhang *et al*., 2014). In the present research, only three deletions were larger than 10 bp, similar to the previous studies (Char *et al*., 2017; Ma *et al*., 2015; Zhang *et al*., 2014). The deletion of large chromosomal segments with CRISPR/Cas9 have been reported in rice and non‐plant species (Xiao *et al*., 2013; Zhou *et al*., 2014). In our study, deletion between two target sites were also detected, which proved that CRISPR/Cas9 with two sgRNAs targeting two distantly spaced target sites can be used to remove gene clusters or induce large genome deletions in switchgrass. More interestingly, this large deletion is observed in plants derived from independent transgenic events (plants 30‐1, 30‐3, 35‐2 and 90‐3 for the *tb1a* gene) and for different genes (35‐2 for both *tb1a* and *tb1b*).

Effective creation and detection of mutant allele in a polyploid switchgrass are critically important. The switchgrass cultivar used in the present study, Alamo, is a tetraploid and is highly heterozygous due to cross‐pollination in nature; therefore, each seed may represent a different genotype. To ensure observed sequence variation are indeed mutations induced by CRISPR/Cas9, rather than natural allelic variation, each callus line derived from a single caryopsis was strictly kept separate. Thus, each callus line represents a distinct genotype and sequences from mutants derived from one callus line are compared only with the wild type derived from the same callus line. Surprisingly, individual plants regenerated from the same callus line may carry different mutations as is shown in plants 35‐2 and 35‐3. This suggests that CRISPR/Cas9 acts continuously and independently on different alleles. Enrichment of mutant alleles with restriction enzyme digestion that removes non‐mutant alleles before PCR amplification is critically important to detect mutant alleles when they are present in low copies. For *tb1* genes, for example, without allele enrichment, among 40 transgenic plants, only twelve plants with mutations in *tb1a* and two plants with mutations in *tb1b* were identified, whereas with allele enrichment approach, all of the 40 transgenic plants were shown to contain *tb1a* mutant alleles and five transgenic plants contained *tb1b* mutant alleles. Thus, for polyploid plants, allele enrichment with restriction enzyme is a highly effective method of detecting mutants induced by CRISPR/Cas9.

The generation of a homozygous mutant for the *PGM* gene suggests that CRISPR/Cas9 can act continuously until all four alleles are mutated at the target site. It also suggests that CRISPR/Cas9 acted early on in the transformation process so all cells of the transgenic embryogenic calli are homogenous, carrying the same mutation. The ability to produce homozygous mutant plants in T0 generation is of great importance, particularly for self‐incompatible perennial grasses, as this will sidestep the need of crossing genetically unrelated heterozygous mutants to generate mutants homozygous for the gene of interest. Furthermore, such homozygous mutants will unlikely exhibit inbreeding depression that is typical of inbred lines of outcrossing species because they are homozygous at the targeted sites only. Generation of such homozygous mutants in switchgrass or other perennial grasses with self‐incompatibility and high ploidy levels will greatly facilitate characterization of gene function in such species. The mechanism of producing same allelic mutation is still not clear. Based on the types of mutation observed for the *tb1* genes, independent, identical allelic mutations are not common in switchgrass, which is similar to the previous research in rice (Ma *et al*., 2015). We did not observe any discernible phenotypic changes in the *PGM* homozygous mutants. Because there are two *PGM* genes with 90% similarity in switchgrass, it is likely that these two genes are functionally redundant, making it impossible to observe the phenotypic changes if only one gene is mutated. In that case, double mutants would need to be made.

There is a mismatch between the sgRNA sequence used for mutagenesis and the target sequence in *PGM* (A/G, 18 bp from the PAM sequence, Figure 2a), but mutations were still successfully recovered from the callus lines. The sgRNA for the second target site in the *tb1* genes also has one nucleotide mismatch with the *tb1b* target region that is at the 12th position distal to PAM sequence; however, mutations were still induced by CRISPR/Cas9. Taken together, apparently in switchgrass, CRISPR/Cas9 is able to recognize alleles with one nucleotide mismatch and still produce mutations. This result is similar to the previous studies in which single or multiple mismatches within the target region do not completely prevent CRISPR/Cas9 targeting as long as the mismatches are present outside the seed sequence (Hsu *et al*., 2013; Semenova *et al*., 2011).

sgRNA feature plays important roles for CRISPR/Cas9 targeting efficiency. In Arabidopsis, it has been suggested that the expression level of sgRNA might be the limiting factor for CRISPR/Cas9 function (Ma *et al*., 2015). The secondary structure of the sgRNA which is dependent on the GC content within the sequence also plays an important role in CRISPR/Cas9 targeting (Ma *et al*., 2015). In our study, these sgRNAs that generated mutations have a GC content ranging from 60% to 70%. It has been reported that targets with higher GC contents have relatively higher editing efficiencies (Ma *et al*., 2015). However, recently it has been reported that sgRNAs activity assessed in transient assay has a low correlation with bioinformatics prediction in wheat (Wang *et al*., 2016). More research is needed to determine whether there is any correlation between the sgRNA features and targeted mutagenesis efficiency by CRISPR/Cas9 in switchgrass.

Tiller density has been shown to have a consistently large effect on biomass yield in upland switchgrass (Boe and Beck, 2008). Tillers result from the outgrowth of axillary buds, which are normally in dormancy in species with a strong apical dominance. The outgrowth of axillary buds is determined by both intrinsic genetic factors as well as external environmental cues such as shade (Sarath *et al*., 2014). *tb1* is one of the best studied regulator of shoot branching, and homologs of *tb1* have similar functions in other grasses (Doebley *et al*., 1997). Earlier studies in maize have shown that *tb1* gene functions in a dosage‐dependent manner; therefore, phenotypic changes can even be observed in non‐homozygous mutants. With CRISPR/Cas9 system, we successfully mutated the two *tb1* genes simultaneously in switchgrass. Individuals with *tb1* gene mutations increased tiller numbers with varying degrees. These mutants are valuable material for developing switchgrass cultivars with high biomass yield because other agronomic traits do not appear to be compromised.

## Conclusions

In summary, we first demonstrated that CRISPR/Cas9 is able to mediate targeted mutagenesis in the tetraploid switchgrass cultivar ‘Alamo’. The mutation efficiency varies from 11% for *tb1b* to 95.6% for *tb1a*. Enrichment of mutant alleles by restriction enzyme digestion is important for detecting CRISPR/Cas9‐induced mutation in switchgrass. In addition, CRISPR/Cas9 can be used for multiplex genome editing in switchgrass by simultaneously editing two genes. Individual plants derived from the same callus line may contain different mutations. Finally, the production of homozygous mutant for the target gene, *PGM* in T0 generation without inbreeding, shows great potential for gene functional analysis and germplasm improvement in switchgrass as well as in other self‐incompatible perennial grasses with high ploidy levels. The transient assay protocol that we developed for switchgrass is a valuable tool that can be used to test different CRISPR/Cas9 with various target sequences.

## Materials and methods

### Protoplast isolation and transient gene expression

Leaves of 9‐ to 14‐day‐old plants of switchgrass cultivar ‘Alamo’ grown in potting soil were used for protoplast isolation following the protocol by Mazarei *et al*. (2008) with modifications. Briefly, leaves were cut into 0.5‐ to 1‐mm‐long segments on filter papers followed by enzyme digestion with a 10‐ml enzyme solution [0.6 m mannitol, 10 mm MES (pH 5.7), 1.5% cellulose (Onozuka R‐10), 0.75% macerozyme R‐10, 0.1% BSA, 1 mm CaCl_2_, 5 mm β‐mercaptoethanol] for 6–8 h with gentle shaking at 40–50 r.p.m. under dark. The solution was filtered through a 40‐μm nylon mesh filter. Protoplasts were washed two times with one volume of W5 washing buffer [154 mm NaCl, 125 mm CaCl_2_, 5 mm KCl, 2 mm MES (pH5.7)] and were collected by centrifugation at 250 ***g*** for 3 min and resuspended in 200 μL of MMG solution [0.6 m mannitol, 15 mm MgCl_2_, 4 mm MES (pH 5.7)]. To examine if the protoplasts can be successfully used for transgene expression, we transfected ~3 × 10^4^ protoplasts with 20 μg plasmid DNA carrying the *GFP* gene driven by the rice ubiquitin gene promoter using the PEG‐mediated DNA uptake method. GFP signals are observed with a NIKON ECLIPSE E200 microscope.

### Gene isolation

The genes *tb1a*,* tb1b* and *PGM* were isolated and sequenced using gene‐specific primers (Table S2) based on the corresponding annotated genes in Phytozome (www.phytozome.net) with the following thermocycler settings: initial denaturing at 98 °C for 5 min, 30 cycles of denaturing at 98 °C for 30 s, annealing at 55 °C (*tb1a* and *tb1b*) or 60 °C (*PGM*) for 30 s, extension at 72 °C for 1 min, then a final extension at 72 °C for 5 min. Sanger sequencing was carried out at the Iowa State University DNA facility (http://www.dna.iastate.edu/) using the Applied Biosystems 3730xl DNA Analyzer. The results were aligned with the sequences obtained from Phytozome using the BLAST tool from the NCBI webpage http://www.ncbi.nlm.nih.gov/ (Sayers *et al*., 2011).

### Selection of target sequences and construction of CRISPR/Cas9 binary vector

Oligos of 19–21 nucleotides were chosen manually from within exon regions of the isolated genes and were synthesized by Integrated DNA Technology (Coralville, IA) (Table S1). The CRISPR/Cas9 constructs were assembled using Gateway^®^ cloning technology (Hartley *et al*., 2000) using the destination vector pUbi‐Cas9 and entry vector (pgRNA4) (Figure 1a). The entry vector was first linearized by either the restriction enzyme *Bsa*I or *Btg*ZI, and then the dsDNA oligos were ligated into the linearized entry vector. The entry vector plasmid DNA containing the guide strand(s) (entry:guide) was transformed into competent *E. coli* DH5α cells. Colony PCR was used to confirm the correct insertion (Table S2). Thermocycler settings were initial denaturing temperature at 98 °C for 5 min, 30 cycles of denaturing at 98 °C for 30 s, annealing at 55 °C for 30 s, extension at 72 °C for 1 min, then a final extension at 72 °C for 5 min. A single colony was then propagated by culturing in liquid LB medium, and the plasmid DNA was extracted using Mini Plasmid Kit from IBI Scientific (Peosta, IA).

Through Gateway LR recombination reaction with LR Clonase II (Invitrogen Carlsbad, CA), the sgRNA expression cassettes were placed into the destination vector with the rice codon‐optimized Cas9 gene driven by maize ubiquitin promoter. The LR reaction products were again transferred, selected and propagated in *E. coli* DH5α in the same manner as the entry:guide plasmid. After that, plasmid DNAs of various CRISPR/Cas9 constructs were transferred into *Agrobacterium* strain C58C1 for plant transformation experiments.

### Plant materials and generation of transgenic plants

Mature caryopses of the lowland switchgrass cultivar ‘Alamo’ (2n = 4x = 36) were surface‐sterilized in 100% chloride for 2 h and rinsed three times with sterilized water, and then placed in callus induction medium containing MS basal salts and B5 vitamins, 30 g/L maltose, 4 mg/L 2,4‐D, 0.8 mg/L 6‐benzylaminopurine (BAP) and 2 g/L Phytagel. Embryogenic calli were produced from caryopses 6–12 weeks after culture under dark at 25 °C. Type II embryogenic callus (Figure S3) was used for all plant transformation experiments (Burris *et al*., 2009). As embryogenic calli were formed, they were kept together and propagated with callus pieces produced from the same caryopsis forming callus lines of identical genetic backgrounds. Calli were subcultured every 3–4 weeks on maintenance medium (callus induction medium supplemented with 1.6 mg/L L‐proline).

Prior to genetic transformation via *Agrobacterium*, embryogenic calli were subcultured onto fresh maintenance medium 10 days beforehand to ensure calli were actively growing. *Agrobacterium tumefaciens* strain C58C1 carrying different binary vectors was used to transform different callus lines. *Agrobacterium* C58C1 was prepared in liquid medium solution containing 200 μm Acetosyringone, which increases the effectiveness of *Agrobacterium* infection (Sheikholeslam and Weeks, 1987). The calli were then submerged in this solution in a covered Petri plate and placed on a shaker at 75 r.p.m. at room temperature for 10 min. The calli were removed and placed on top of five stacked sterilized filter papers within a Petri dish. It was sealed and placed in the dark for 3 days at 24 °C for desiccation treatment. Calli were then moved to a resting medium for 3–7 days.

Calli from the resting medium were transferred to the selection medium containing 100 mg/L hygromycin for 4–6 weeks. Actively growing calli (Figure S4) were moved onto regeneration medium and placed in a growth chamber with a light intensity of 140 μm/m^2^/s at a photoperiod of 16‐/8‐h light/dark cycle and a temperature of 25 °C (Figure S5) (Li and Qu, 2011). After shoots produced roots, plantlets were moved to a mist room for acclimation for 7–10 days. Plants were grown in a commercial soil mix (Sunshine soil mix #1, Sun Gro Agawam, MA) of peat moss and perlite and maintained at 23 °C in the glasshouse with a 16‐/8‐h (day/night) photoperiod with a light intensity of approximately 400 μm/m^2^/s. From each putative transgenic event, at least two plants were further confirmed by PCR amplification with primers designed based either on the sgRNA sequence in combination with a primer based on the Cas9 promoter, or on the hygromycin resistance *hpt* gene (Table S2).

### Characterization of transgenic plants

All transgenic events were characterized by DNA sequencing. DNA was extracted either from leaf tissue with the CTAB method (Murray and Thompson, 1980). A PCR screening was performed to verify the insertion of the CRISPR transgene before further characterization with primers specific to the sequence of Cas9/sgRNA vector (Table S1). Thermocycler settings were initial denaturing temperature at 98 °C for 5 min, then 30 cycles with 98 °C denaturing for 30 s, 55 °C annealing for 30 s, 72 °C extension for 1 min, then a final extension of 5 min.

The relevant regions of target genes were PCR‐amplified using site‐specific primers (Table S2). The thermocycler settings were as follows: initial denaturing temperature at 98 °C for 5 min, then 30 cycles with 98 °C for 30 s, 55 °C (for *tb1a* and *tb1b*) or 60 °C (for *PGM*) for 30 s, 72 °C for 1 min, then a final extension of 5 min. The PCR products used for sequencing were treated with ExoSAP‐IT (Applied Biosystems, Foster City, CA) before sequencing. Sequence results were aligned against the sequence of the untransformed control to identify InDels, and chromatograms were examined for double peaks that could indicate a heterozygous insertion/deletion.

If sequencing of PCR products showed double or multiple peaks in the chromatogram, the amplicons were subjected to cloning and a number of clones were subjected to sequencing to verify putative mutations and to determine the exact nature of InDels. Similarly, the relevant regions were PCR‐amplified and treated with ExoSAP‐IT. The PCR product was then ligated into pGEM^®^‐T Easy vector from Promega (Madison, WI) for 16 h at 4 °C. The *E. coli* strain DH5α was transformed with the pGEM^®^‐T Easy vector by heat shock and incubated with SOC medium. After incubation, the mixture was plated onto LB medium containing 100 mg/L ampicillin, 1 mm IPTG and 200 mg/L X‐Gal for propagation and blue/white screening. White colonies were propagated in 5 mL liquid LB medium containing 5 μL ampicillin for 16 h at 37 °C in a shaker at 150 r.p.m. Plasmid extraction was carried out using the Mini Plasmid Kit from IBI Scientific, and the plasmid was sequenced to confirm the mutation in the allele.

### Phenotype analysis

Five transgenic plants with CRISPR/Cas9‐induced mutations, one transgenic plant without mutation and three wild‐type plants were examined for morphological alteration. Tiller number, stem diameter and plant height were measured at the reproductive stage (Moore *et al*., 1991). Plant height was determined by measuring the length of the tallest tiller for each plant. Average tiller diameter was determined by measuring the diameter of the middle internode for each tiller, and three largest tillers were chosen for measurement. SAS (SAS Institute Inc., Cary, NC) was used to conduct ANOVA and Tukey's range test (Tukey, 1949) was used to determine if there are statistical differences for measured traits between mutant plants and the wild‐type plants.

## Supporting information


**Figure S1** Illustration of the entry vector pENTR4:gRNA4.

**Figure S2** Illustration of the destination vector pUbi‐Cas9.

**Figure S3** Embryogenic calli are induced on Murashige and Skoog (MS) medium for 6–12 weeks. For subsequent propagation, actively growing calli are subcultured on maintenance medium which contains 2 g/L L‐proline. Large pieces are divided into smaller pieces during subculture which lasts for 3–4 weeks before being subcultured again.

**Figure S4** Resistant embryogenic callus (arrows) are selected on selection medium which contains 100 mg/L hygromycin.

**Figure S5** Resistant embryogenic callus regenerated on regeneration medium which contains 50 mg/L hygromycin.

**Table S1** sgRNA sequence for *GFP*,* tb1* and *PGM* genes.
**Table S2** Sequences of primers used for each gene.
