# Supplementary figures and images for: Targeted mutagenesis in tetraploid switchgrass (Panicum virgatum L.) using CRISPR/Cas9
Source: Plant Biotechnol J. 2017 Aug 1;16(2):381–93. doi: 10.1111/pbi.12778 (PMC5787850; doi:10.1111/pbi.12778)

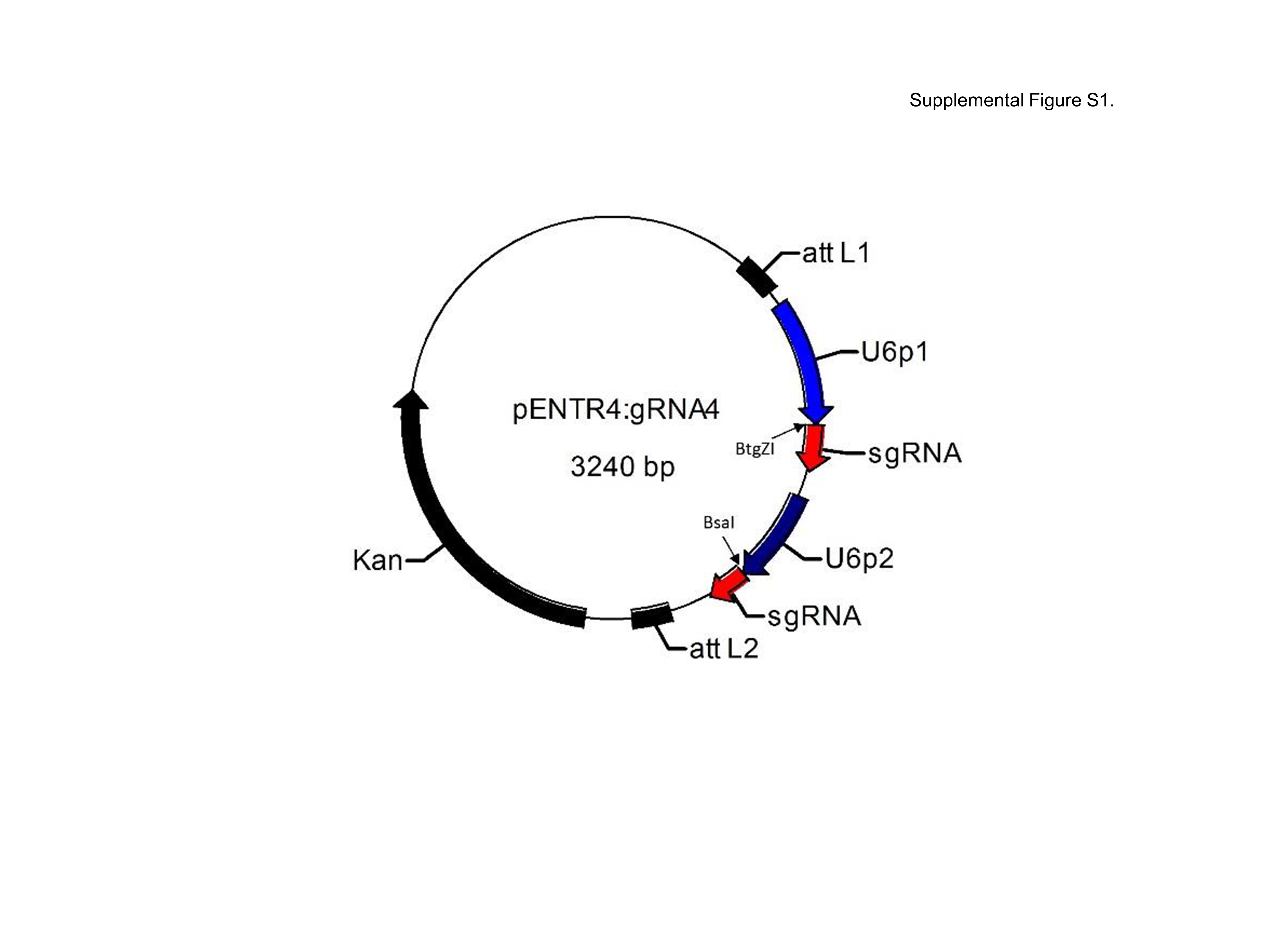

Supplement: Supplementary file 1 — Figure S1 Illustration of the entry vector pENTR4:gRNA4. [file PBI-16-381-s005.tif]

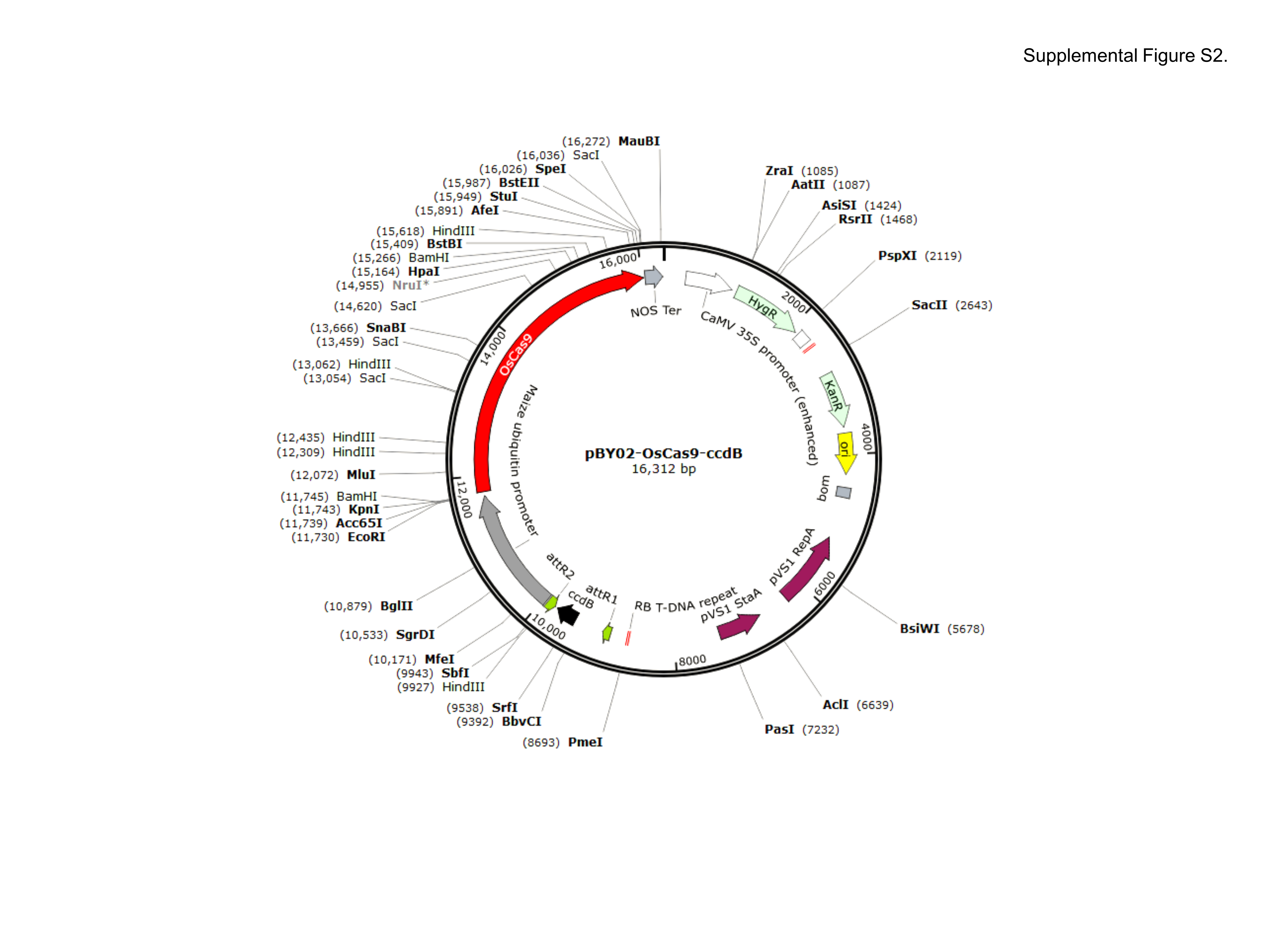

Supplement: Supplementary file 2 — Figure S2 Illustration of the destination vector pUbi‐Cas9. [file PBI-16-381-s004.tif]

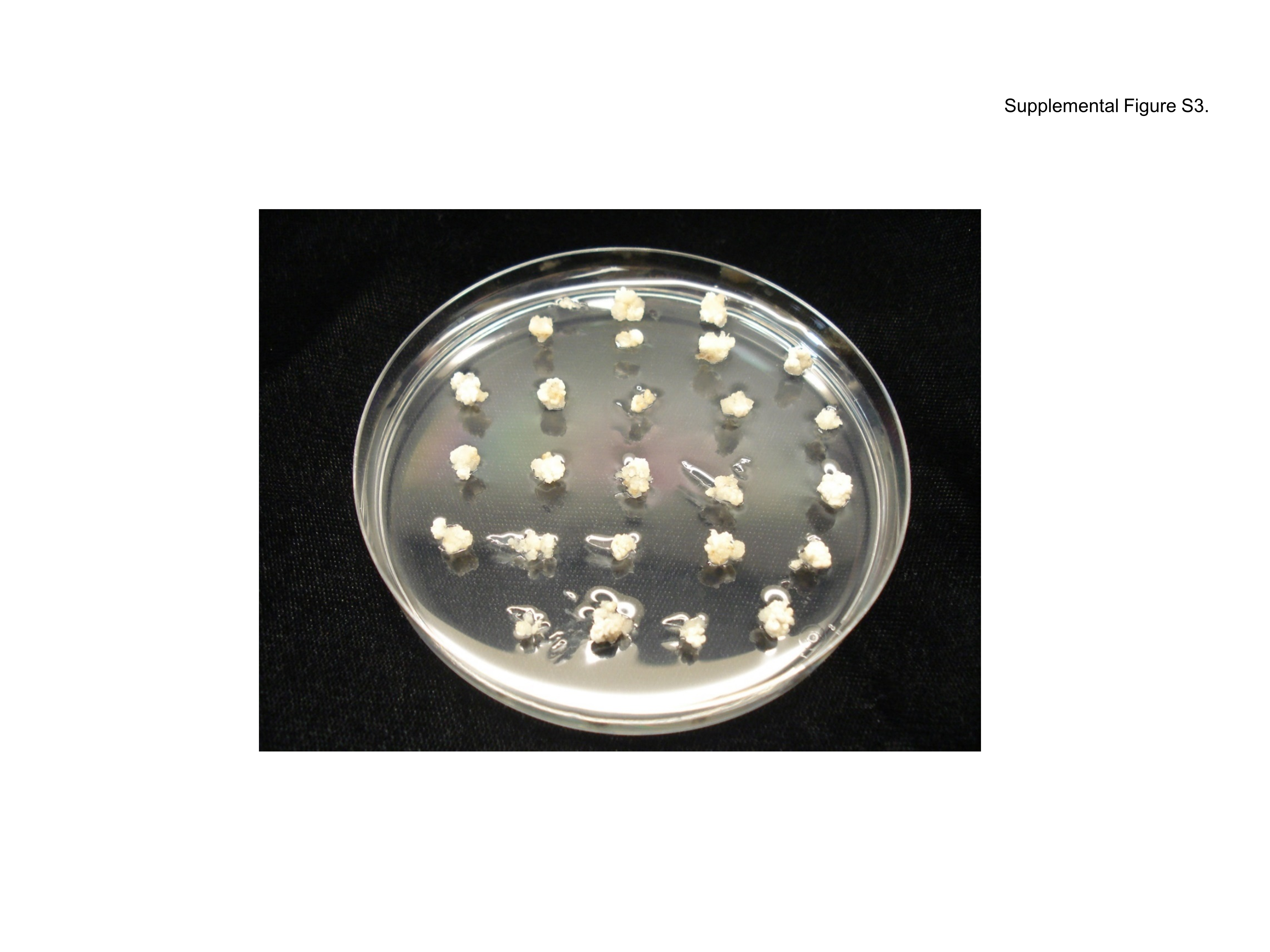

Supplement: Supplementary file 3 — Figure S3 Embryogenic calli are induced on Murashige and Skoog (MS) medium for 6–12 weeks. For subsequent propagation, actively growing calli are subcultured on maintenance medium which contains 2 g/L L‐proline. Large pieces are divided into smaller pieces during subculture which lasts for 3–4 weeks before being subcultured again. [file PBI-16-381-s003.tif]

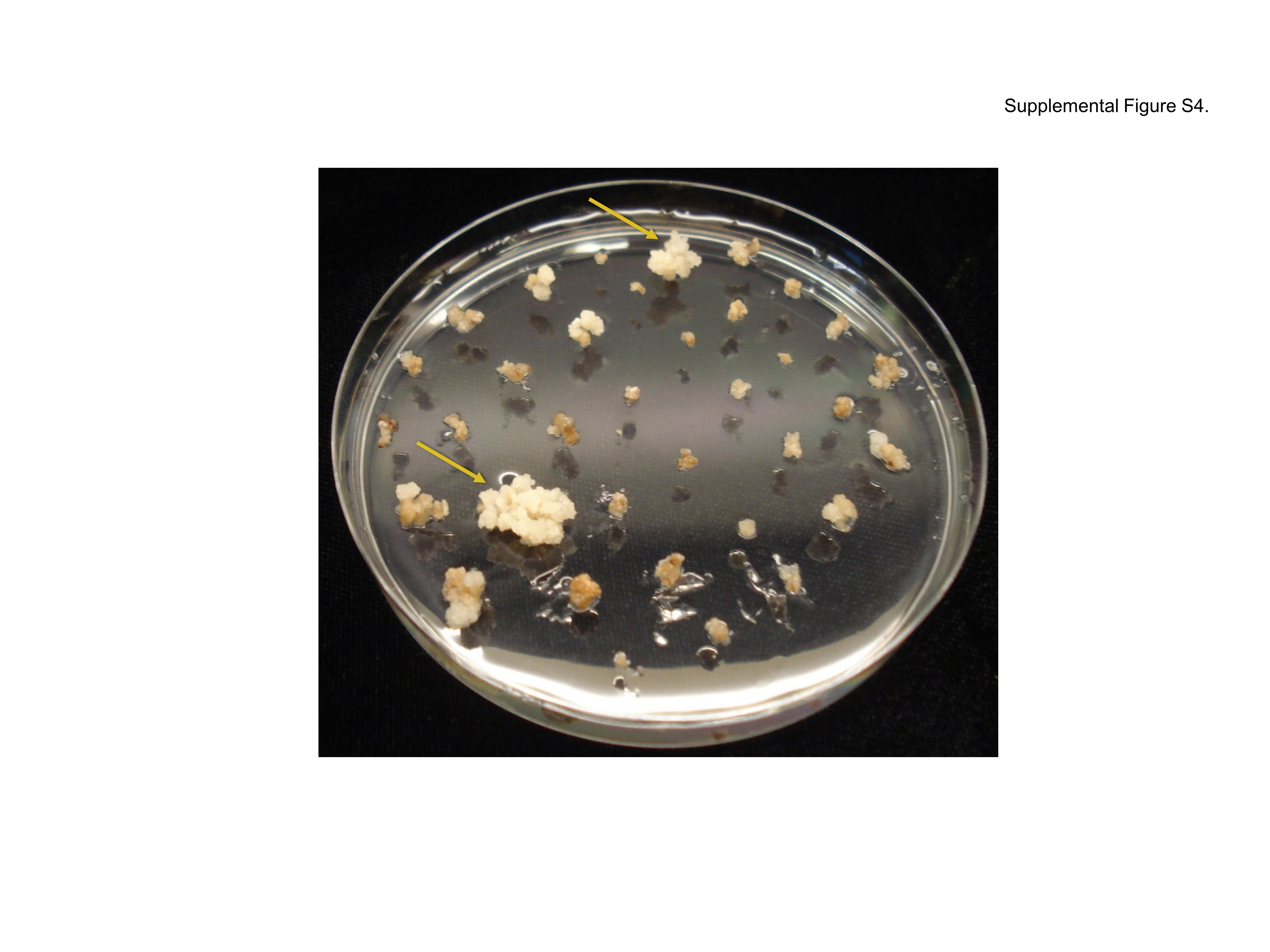

Supplement: Supplementary file 4 — Figure S4 Resistant embryogenic callus (arrows) are selected on selection medium which contains 100 mg/L hygromycin. [file PBI-16-381-s006.tif]

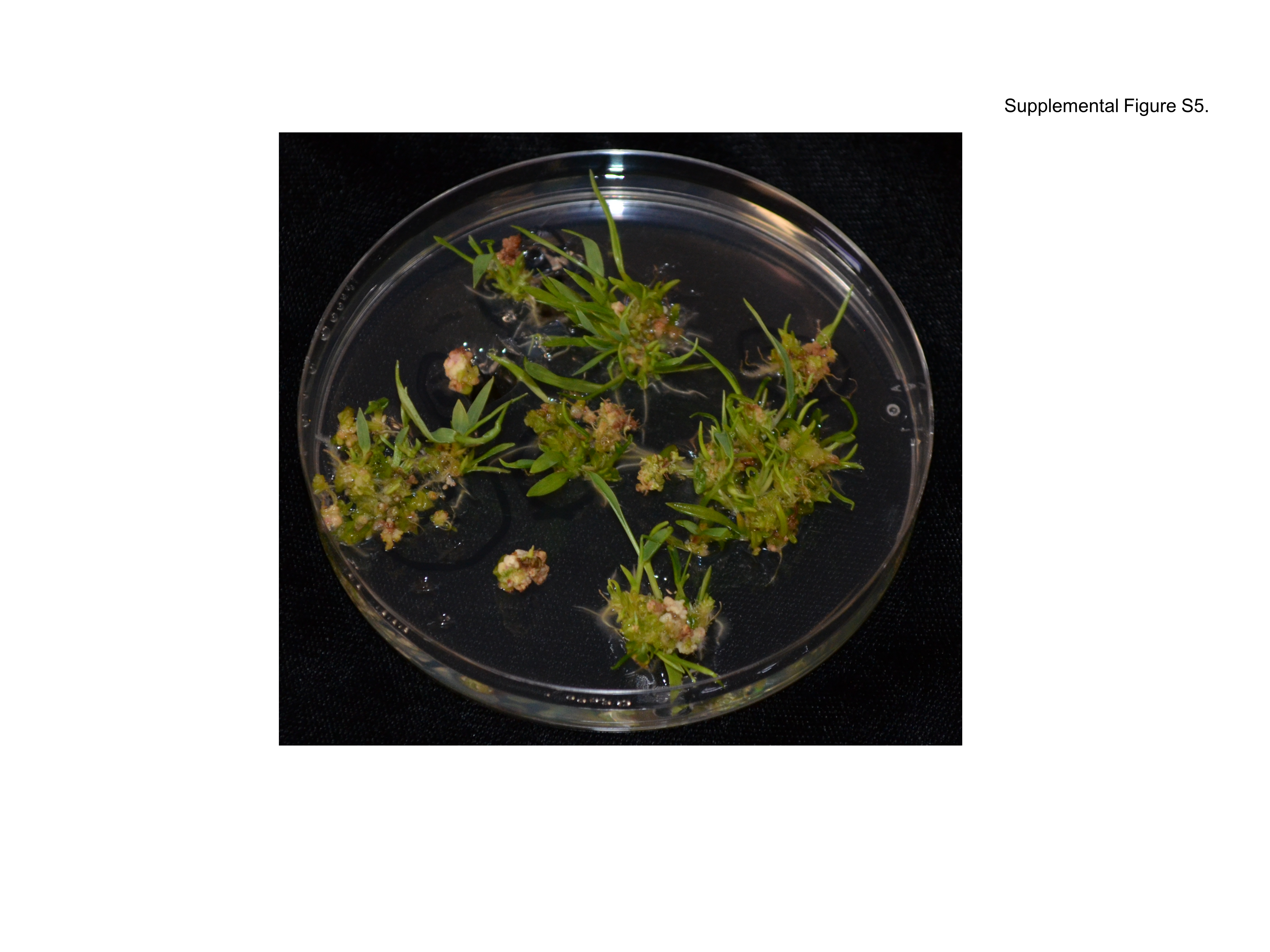

Supplement: Supplementary file 5 — Figure S5 Resistant embryogenic callus regenerated on regeneration medium which contains 50 mg/L hygromycin. [file PBI-16-381-s002.tif]
