# Supplementary material for: Targeted mutagenesis in tetraploid switchgrass (Panicum virgatum L.) using CRISPR/Cas9
Source: Plant Biotechnol J. 2017 Aug 1;16(2):381–93. doi: 10.1111/pbi.12778 (PMC5787850; doi:10.1111/pbi.12778)
Supplement: Supplementary file 6 — Table S1 sgRNA sequence for GFP, tb1 and PGM genes. Table S2 Sequences of primers used for each gene. [file PBI-16-381-s001.docx]

| Genes | Construct | Insertion site | Target sequence (5’—3’) |
| --- | --- | --- | --- |
| Nonfunctional *GFP* | pU6:gRNA_GFP | BtgzI | GACCATGGTGAGCAAGGGGCG |
| *tb1* | pU6:gRNA_TB1 ×2/Cas9 | BsaI | GGTAAAGCGGTAAGTCCATG |
|  |  | BtgzI | GACCGAGCTGGTAGCTGAGG |
| *PGM* | pU6:gRNA_PGM/Cas9 | BsaI | GCACGGAGCTGGTGGTGGTG |

Table S1. sgRNA sequence for *GFP*, *tb1* and *PGM* genes

Table S2. Sequences of primers used for each gene

| **For gene sequencing** | | |
| --- | --- | --- |
| Gene | Forward Primer | Reverse Primer |
| *tb1a* | TGCCGCTCTCTCACATTCAC | GTGCATATCTTGCTGTGCCG |
| *tb1b* | CTTAGTGGCAGGACCTAGCG | AGTTCAACATCACGCGGTCT |
| *PGM* | CATTCCAGGAGTCTGCAACA | AGGACGCTGCTGCTATCATT |
| **For vector validation** | | |
| *tb1* (1^st^ site) | GGCGAGAGAAGCCTAGTGTG | AAACCATGGACTTACCGCTTTACC |
| *PGM* |  | AAACCACCACCACCAGCTCCGTGC |
| **Entry:guide colony PCR, BtgZI** | | |
| *tb1*(2^nd^ site) | TGTTGACCGAGCTGGTAGCTGAGG | AAACCATGGACTTACCGCTTTACC |
| **Transgenic plants confirmation** | | |
|  | **sgRNA (forward)** | **OsCas9 promoter (reverse)** |
| *tb1* | GTGTGGTAAAGCGGTAAGTCCATG | CCTGTTGTCAAAATACTCAA |
| *PGM* | GTGTGCACGGAGCTGGTGGTGGTG |  |
| **Transgenic plant confirmation, *hpt* gene** | | |
|  | Forward Primer | Reverse Primer |
| *All* | GCGAAGAATCTCGTGCTTTC | TCTACACAGCCATCGGTCCAG |
